# Correlation between β-amyloid deposits revealed by BF-227-PET imaging and brain atrophy detected by voxel-based morphometry-MR imaging: a pilot study

**DOI:** 10.1097/MNM.0000000000001042

**Published:** 2019-06-25

**Authors:** Nobuhisa Maeno

**Affiliations:** Department of Health Science, Faculty of Medical Welfare, Aichi Shukutoku University, Japan

**Keywords:** BF-227-PET, local β-amyloid deposition, local brain atrophy, Alzheimer’s disease

## Abstract

**Objective:**

The purpose of this study was to investigate whether β-amyloid (Aβ) deposition was associated with local atrophy of corresponding areas in the brain.

**Methods:**

[11C]2-[2-(2-Dimethylaminothiazol-5-yl) ethenyl-6-[2-(fluoro)ethoxy]benzoxazole (BF-227)-PET, MRI and neuropsychological tests were carried out on 56 subjects, out of which 21 were patients with Alzheimer’s disease (AD), 20 were patients with mild cognitive impairment (MCI) and 15 were normal controls (NC). The BF-227 uptake in each local brain region was set up with automated anatomical labeling atlas using Wake Forest University PickAtlas software and local standardized uptake value ratios of BF-227 were calculated as the average value of right and left using the MRIcron software.

**Results:**

Group comparisons of Aβ deposition as determined by BF-227 uptake using PET imaging showed no significant differences between MCI and AD. Aβ deposition was significantly higher in MCI and AD than in NC. The correlation analysis between local Aβ deposition and gray matter atrophy showed that in AD, the Aβ deposition in the inferior temporal gyrus was strongly related to the gray matter atrophy in this region. On the contrary, the Aβ deposition in the precuneus was associated with the atrophy in the right occipital-temporal region. In the NC, the Aβ deposition in the inferior temporal gyrus was associated with the atrophy in the precuneus.

**Conclusion:**

In the AD, the relationship between the Aβ deposition and local atrophy is area-dependent. In NC, Aβ deposition in the inferior temporal gyrus correlated to the atrophy in the precuneus.

## Introduction

The deposition of β-amyloid (Aβ) in the brain of patients with Alzheimer’s disease (AD) is closely related to pathological findings such as appearance of senile plaques, neurofibrillary tangle formation and neurodegeneration [1]. The amyloid hypothesis is one of the leading hypotheses to explain the mechanism of AD onset [2].

However, as senile plaque formation and neuronal loss are temporally and spatially discordant in some cases, it was concluded that senile plaque formation is not indispensable for neurodegeneration [3].

Recently, amyloid imaging has been used to visualize the deposition of Aβ in the brain by PET. [^11^C]2-[2-(2-Dimethylaminothiazol-5-yl)ethenyl-6-[2-(fluoro)ethoxy]benzoxazole (^11^C-BF-227, BF-227), an amyloid imaging PET probe developed at Tohoku University research center, has a relatively high efficiency in crossing blood-brain barrier (BBB), high binding affinity for Aβ aggregates and high specificity for fibrillary Aβ deposits [4]. Moreover, the effectiveness of this probe in diagnosing AD has been suggested by a clinical exploratory trial performed in this center [5]. Ever since, several studies have explored the utility of BF-227. Kudo *et al*. [5] observed that the mean standardized uptake value (SUV) ratio of BF-227 uptake in the frontal, lateral temporal, parietal, temporooccipital, occipital, anterior/posterior cingulate cortices and striatum was significantly greater in patients with AD than in normal elderly subjects. Furukawa *et al*. [6] reported that patients with AD showed increased BF-227 uptake in neocortical areas and striatum as well as decreased glucose metabolism in temporoparietal, posterior cingulate and medial temporal areas. Moreover, mild cognitive impairment (MCI) patients showed a significant increase in BF-227 uptake in neocortical areas similar to AD, and the most significant difference of BF-227 uptake was observed in the parietal lobe. Neocortical BF-227 uptake negatively correlated with glucose metabolism. These results suggest that uptake of BF-227 often happens in the cerebral cortex.

Most studies investigating the association between Aβ deposition and brain atrophy have been performed using Pittsburgh compound B (PiB)-PET, and can be summarized as follows: Chételat *et al*. [7] reported that association between whole brain atrophy and local brain atrophy with Aβ deposition was observed only in patients with AD showing subjective cognitive impairment (SCI) but not in normal controls (NC, MCI) and AD patients with MCI. Archer *et al*. [8] reported that in mild and moderate AD, the atrophy rate in the anterior/posterior cingulate gyrus was associated with Aβ deposition.

By contrast, Jack *et al*. [9] reported that brain atrophy was independent from PiB retention in the NC, MCI and AD (amyloid deposition proceeded at a constant slow rate while neurodegeneration accelerated). Driscoll *et al*. [10] reported that in the longitudinal study of NC, no association was detected between PiB retention and the development of the brain atrophy. In the results of these two above-mentioned PiB reports, as for the mean neocortical PiB-SUVR value of AD, it seems that little has been reported on the relation with the cerebrocortical atrophy.

Using the BF-227-PET on NC, MCI (nonconverter and converter) and AD, Waragai *et al*. [11] reported no significant correlation between BF-227 uptake and whole brain or parahippocampal gyrus volume.

These reports have been related to only the stages preceding the conversion to AD (preclinical stages of AD), but not all stages of AD development or normal elderly subjects. The results obtained so far are controversial, and the relationship between the deposition of senile plaques (Aβ) and brain atrophy in the development of AD remains unclear. Besides, little has been reported about the effect of local Aβ deposition on gray matter atrophy, as well as the study using BF-227. To our knowledge, Becker *et al*. [12] used functional MRI (fMRI) and reported that decreased cerebral cortex thickness of the precuneus extending into the posterior cingulate gyrus was due to Aβ deposition, and that this decrease preceded clinical symptoms. In addition, Bourgeat *et al*. [13] reported that significant correlation was found between PIB retention in the inferior temporal areas and hippocampal volume in the PIB-positive healthy control. It would be interesting to determine whether Aβ deposition in the precuneus or inferior temporal gyrus is associated with atrophy in other areas of the brain. Results provided by the study using BF-227 were insufficient. It is known that BF-227 labels mature senile plaques more efficiently than PiB retention [12]. As mentioned above, it is considered that BF-227 uptake by the cerebral cortex is more extensive than that of PiB. It would be interesting to establish whether BF-227 uptake pattern differs from that of PiB retention in the NC, MCI and AD patients.

In this study, we performed a clinical trial by PET imaging of amyloid using BF-227 in NC, MCI and AD subjects to elucidate the differences between the distribution patterns of Aβ deposition in these groups. In addition, we compared local brain atrophy levels among the three groups using voxel-based morphometry (VBM)-MR images. Taken together, these two methodologies should clearly demonstrate the relationship between local Aβ deposition and gray matter volume loss.

## Methods

### Subjects

Fifty-six individuals were subjected to BF-227-PET, MRI and neuropsychological tests at Tohoku University, out of which 21 were patients with AD [mean age = 73.7 ± 6.7 years, 14 females, mean mini-mental state examination (MMSE) score = 20.1 ± 3.4], 20 were patients with MCI (mean age = 76.9 ± 4.7 years, 11 females, mean MMSE = 25.5 ± 2.3) and 15 were controls (NC) (mean age = 65.9 ± 4.9 years, 7 females, mean MMSE = 29.9 ± 0.3) (Table 1).

**Table 1:**
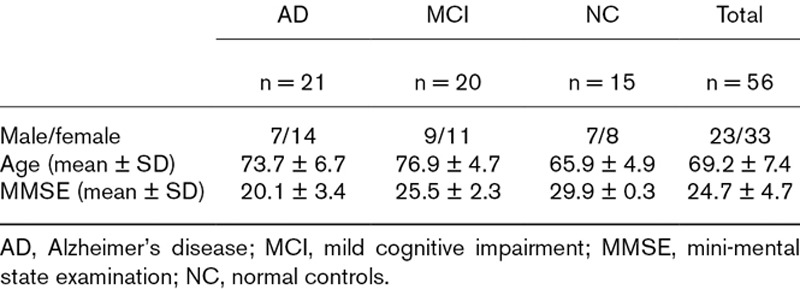
The demographic information for AD, MCI and NC

Patients with AD and MCI were recruited from the Tohoku University Hospital Dementia Patients Registry. The diagnosis of AD was made according to the National Institute of Neurological and Communicative Diseases and Stroke/Alzheimer’s Disease and Related Disorders Association (NINCS-ADRDA) criteria. Criteria for the diagnosis of MCI were those defined by Petersen *et al*. [14]. The NC group consisted of volunteers who were taking no medication and had no cognitive impairment or cerebrovascular lesions detected by MR images. This study was approved by the ethics committee on clinical investigations of Tohoku University School of Medicine, and all participants provided written informed consent.

### Imaging

All subjects underwent BF-227 PET and 3D-MRI. The ^11^C-BF-227 PET study was performed using a SET-2400 W PET scanner (Shimadzu corp. Kyoto, Japan. https://www.shimadzu.com/). BF-227 dynamic PET was performed for 60 min after the injection of 370–740 MBq BF-227. Spatially normalized SUVR images were calculated from static 20–40 min images using the reference values in the cerebellar region of interest (ROI). The BF-227 uptake of each local brain regions was set up with automated anatomical labeling (AAL) atlas in the Wake Forest University (WFU) PickAtlas software version 2.4 (http://fmri.wfubmc.edu/software/PickAtlas) and the local SUVR of BF-227 (ROI) were calculated as the average value of right and left by the MRIcron software (http://www.mccauslandcenter.sc.edu/mricro/mricron/). The WFU PickAtlas software was operated on Matrix Laboratory (MATLAB) version 2009b (http://www.mathworks.co.jp/). The T1-weighted MR images were obtained using a 1.5 Tesla machine (GE Signa Hispeed; GE Healthcare corp., Milwaukee, WI, USA. https://www.gehealthcare.com/). To correct the nonuniformity in the intensity of acquired T1-3D-MR images, N3 software was used. Spatial normalization and segmentation were performed by SPM8 (Statistical Parametric Mapping, 2008 Edition) and DARTEL (diffeomorphic anatomical registration through exponentiated Lie Algebra).

### Statistical analyses (the local BF-227 uptake value and imaging)

Statistical Package for the Social Sciences (SPSS) Version 12 was used. The local BF-227 uptake values of the three groups were evaluated using one-way analysis of variance (ANOVA), which was followed by the Bonferroni post hoc test.

We evaluated three detailed analyses as described below:

1. The group comparison of gray matter atrophy using VBM-MRI.
2. The group comparison of Aβ deposition using BF-227.
3. The correlation analysis between local BF-227 uptake value and gray matter atrophy.

The analysis of the VBM-MR images of local brain atrophy areas was performed using local BF-227 uptake values in the ROI into a parameter. That is, this analysis made the local BF-227 uptake value of which was the significant difference of BF-227 uptake value in the AD, MCI, and NC into “covariates” of multiple regression for SPM8. Statistical significance was set at *P* < 0.05 for all analyses.

## Results

Several brain regions such as the precuneus, middle/inferior temporal gyrus, cuneus, superior/middle occipital gyrus, supramarginal gyrus and angular gyrus showed significant differences in local BF-227 uptake value among the three groups, as established using ANOVA. Table 2 shows all the ROI, where significant differences were observed in this study (the Bonferroni post hoc test). Remaining regions did not show significant differences of BF-227 uptake value between the groups.

**Table 2:**
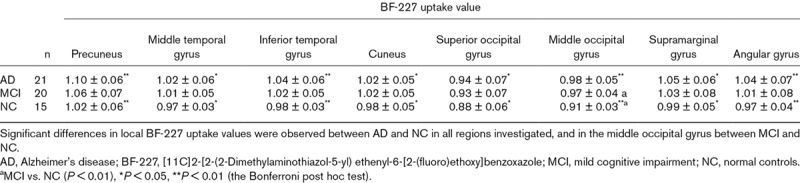
The results of local BF-227 uptake value

Among all regions examined, the highest value of BF-227 uptake was observed in the precuneus in all three groups. A significant difference in local BF-227 uptake values between AD and NC was detected in the regions described above. Significant differences were not observed between MCI and AD or between MCI and NC except for the middle occipital gyrus in the latter case (Table 2).

### Group comparisons of gray matter atrophy using voxel-based morphometry-MRI

**Fig. 1:**
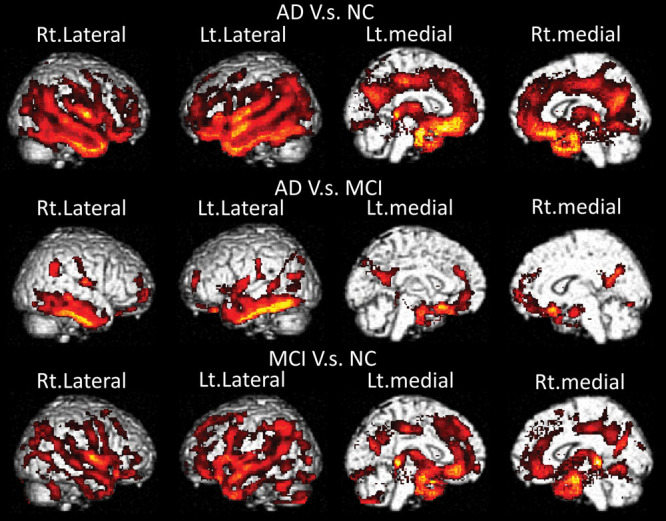
Group comparisons of gray matter atrophy (*P* < 0.05, ext = 100).

Figure 1 shows that significantly lower gray matter volumes in AD compared to NC were widely observed in the anterior/posterior cingulate gyrus, precuneus, inferior frontal gyrus, parieto-occipital areas, medial temporal areas and temporal lobe. AD patients showed local atrophy in the inferior frontal gyrus, orbital areas and posterior cingulate gyrus compared to MCI. MCI patients showed local atrophy in the medial temporal areas and diffuse atrophy in the temporal lobe compared to NC. The gray matter atrophy was observed in the order of AD patients > MCI patients > NC subjects.

### Group comparisons of β-amyloid deposition using BF-227

**Fig. 2:**
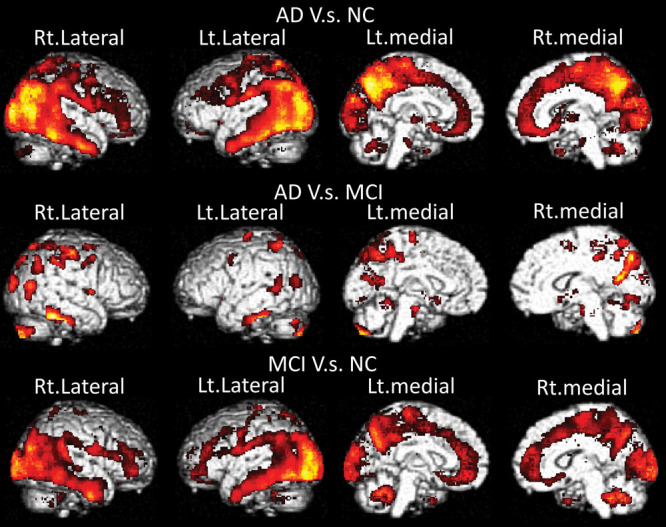
The group comparison of β-amyloid (Aβ) deposition (*P* < 0.05, ext = 100).

Figure 2 shows that significantly higher BF-227 uptake values were observed in the posterior cingulate gyrus, cuneus, precuneus, occipital lobe and inferior temporal gyrus of patients with AD than NC.

No significant differences in BF-227 uptake values were observed between AD and MCI. Patients with MCI showed BF-227 uptake values comparable to those in NC group in the anterior/posterior cingulate gyrus, cuneus, precuneus, occipital lobe and inferior temporal gyrus. The Aβ deposition was already completely full at the MCI stage, so that the increase in Aβ deposition can be shown in the order of AD patients ≒ MCI patients > NC subjects.

### Correlation analyses between local BF-227 uptake and gray matter atrophy

First, the analyses of VBM-MR images of local brain atrophy areas were performed using the precuneus BF-227 uptake values (BF-227 ROI) into a parameter. The reason for choosing the Aβ deposition in the precuneus as a first parameter was that this region showed the highest BF-227 uptake value among all regions (Table 2).

**Fig. 3:**
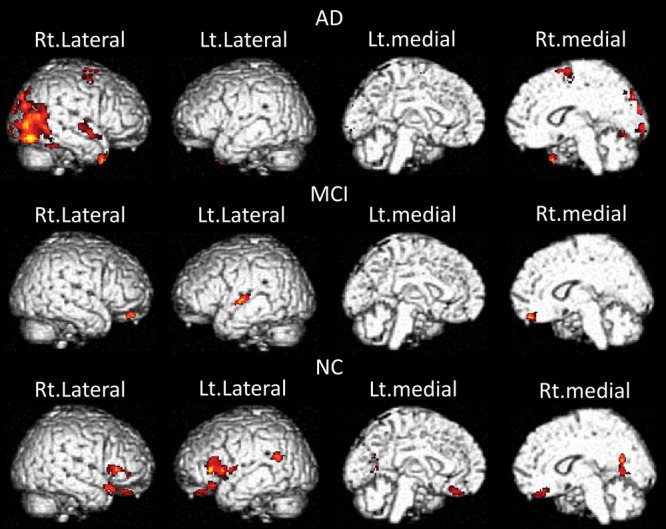
The areas of brain atrophy where significant negative correlation with BF-227 uptake values in precuneus was observed (*P* < 0.05, ext = 100). BF-227, [11C]2-[2-(2-Dimethylaminothiazol-5-yl) ethenyl-6-[2-(fluoro)ethoxy]benzoxazole.

Figure 3 shows that the higher the Aβ deposition in the precuneus, the more extensive was the gray matter volume decrease in the right occipitotemporal area for AD, in the inferior frontal gyrus for NC, while there were no expanding atrophy regions in MCI group.

Second, analyses of the VBM-MR images of local brain atrophy areas were performed using the inferior temporal gyrus BF-227 uptake values into a parameter.

**Fig. 4:**
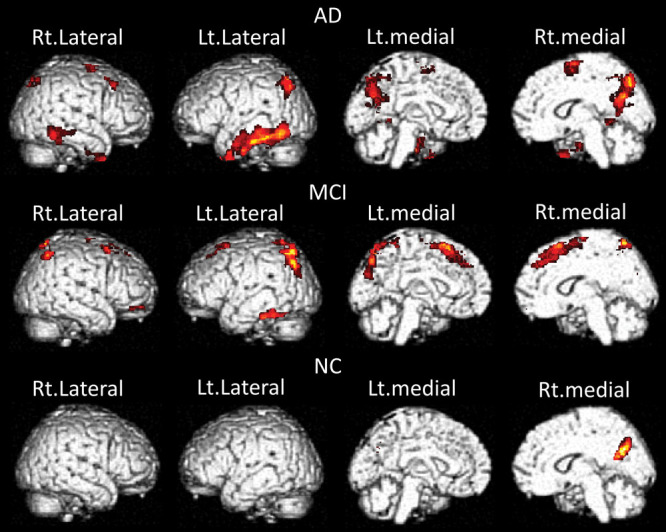
The areas of brain atrophy where significant negative correlation with BF-227 uptake values in inferior temporal gyrus was observed (*P* < 0.05, ext = 100). Similarly, the higher the β-amyloid (Aβ) deposition in the middle temporal gyrus, middle occipital gyrus, supramarginal gyrus and angular gyrus, the more extensive was the gray matter volume decrease, and the affected areas were almost identical to those described for inferior temporal gyrus. However, the relation of Aβ deposition in the supramarginal gyrus was unremarkable to the gray matter atrophy of the right precuneus in normal controls (NC) (data not shown). BF-227, [11C]2-[2-(2-Dimethylaminothiazol-5-yl) ethenyl-6-[2-(fluoro)ethoxy]benzoxazole.

Figure 4 shows that the higher the Aβ deposition in the inferior temporal gyrus, the more extensive was the decrease in the gray matter volume in the precuneus, inferior temporal gyrus and inferior parietal lobe for AD, in the medial frontal gyrus, precuneus and inferior parietal lobe for MCI, and in the right precuneus for NC. Such decrease was therefore relatively local. Similarly, the higher the Aβ deposition in the middle temporal gyrus, middle occipital gyrus, supramarginal gyrus and angular gyrus, the more extensive was the gray matter volume decrease within the regions that were also affected by amyloid deposition in the inferior temporal gyrus. However, the relation between Aβ deposition in the supramarginal gyrus and the gray matter atrophy of the right precuneus was unremarkable in NC (data not shown). In addition, in the case of Aβ deposition in other areas, such as cuneus and superior occipital gyrus, gray matter volume loss was observed in the precuneus and inferior temporal gyrus in the AD, similar to when Aβ deposition was observed in the inferior temporal gyrus. In the case of MCI, there was no meaningful correlation with any regions. For NC, the area showing gray matter volume loss was the right precuneus, as in the case of Aβ deposition in the inferior temporal gyrus (data not shown).

## Discussion

To confirm the association between Aβ deposition and local brain atrophy in the development of AD, we performed correlation analysis using BF-227-PET and MR imaging in NC, MCI and AD. First, the group comparison of gray matter volume by VBM-MR imaging among the three groups demonstrated atrophic changes according to the development of AD in regions such as medial temporal areas, anterior/posterior cingulate gyrus, precuneus, parieto-occipital areas and temporal lobe (Fig. 1). These results generally correspond to the described development of AD [15].

Second, the group comparison of Aβ deposition by BF-227 imaging among the three groups yielded no significant differences between AD and MCI. The BF-227 uptake was significantly different between AD and NC in the posterior cingulate gyrus, cuneus, precuneus, occipital lobe, inferior temporal gyrus, etc. No significant difference of BF-227 uptake was shown between MCI and NC in areas such as the anterior/posterior cingulate gyrus, cuneus, precuneus, occipital lobe and inferior temporal gyrus (Fig. 2). This finding suggests that Aβ deposition already reached a plateau in MCI stage, and it would explain why no significant difference of Aβ deposition was detected between AD and MCI. Waragai *et al*. [10] reported that the combined sample of MCI converters and AD patients showed no significant correlation of BF-227 uptake with clinical signs. However, several studies that employed amyloid PET using [^11^C] PiB have shown significant differences in PiB retention between MCI and AD [16]. PiB is currently the most successful among several amyloid imaging agents, and it is probably efficient in detecting relatively immature amyloid lesions [17].

In contrast, the fluorescence intensity of BF-227 is the highest in the core region of mature amyloid deposits [5]. He Shao *et al*. [18] reported that BF-227-PET images clearly demonstrated an abnormal BF-227 uptake in the posterior association areas in MCI converters and AD, and this technique distinguished MCI converters from MCI nonconverters. In our sample, further studies would be needed to differentiate between MCI converters and MCI nonconverters to AD. And also MMSE and background information of the subject was needed more. Moreover, establishing whether the discrepancy in the results is due to a difference in the severity of AD pathology or different affinities for Aβ between PiB and BF-227 would require further studies including more samples.

Finally, to elucidate the association between Aβ deposition and local gray matter atrophy in the development of AD, we performed correlation analysis between BF-227 uptake and gray matter atrophy in the NC, MCI and AD. The analysis of the VBM-MR imaging of local gray matter atrophy areas was done using the local BF-227 uptake value into a parameter. We particularly focused on precuneus and middle/inferior temporal gyrus because these regions showed significant differences in local BF-227 uptake in the three groups. In addition, our results suggest that Aβ deposition in the precuneus and inferior/middle temporal gyrus (including cuneus, superior/middle occipital gyrus, supramarginal gyrus and singular gyrus) was participating in the local atrophy of these different regions. Namely, the areas where Aβ deposition in the precuneus was higher showed more extensive decrease in the gray matter volume. In AD and NC, the correlation was detected in the right occipitotemporal area and in the inferior frontal gyrus, respectively, while no expanding atrophy region was detected in MCI (Fig. 3). The areas in which higher Aβ deposition in the inferior temporal gyrus was associated with more extensive decrease in the gray matter volume were the precuneus, inferior temporal gyrus and inferior parietal lobe for AD, medial frontal gyrus, precuneus and inferior parietal lobe for MCI, and the right precuneus in NC. These regions showed a relative local restriction (Fig. 4). The atrophy of the inferior temporal gyrus and precuneus may be affected by the Aβ deposition in the inferior/middle temporal gyrus, cuneus, superior/middle occipital gyrus, supramarginal gyrus, singular gyrus, etc. Besides, voxel-based analysis of 2-(1-{6-[(2-(18)F-fluoroethyl)(methyl)amino]-2-naphthyl}ethylidene) malononitrile (FDDNP)-PET showed significantly higher FDDNP binding in inferior temporal gyrus in the AD [19]. Moreover, the above-mentioned Bourgeat *et al*. [13] reported that Aβ deposition in the inferior temporal cortex was associated with hippocampal atrophy. In our study, BF-227 uptake in the inferior temporal gyrus in AD showed negative correlation with gray matter density of the inferior temporal gyrus itself suggesting that local amyloid plaque may have directly influenced local tissue atrophy. In other words, it has been suggested that amyloid fibrils cause apoptosis [2]. However, it should be considered that the correlation with the atrophy of the precuneus and the inferior parietal lobe could simply reflect the AD development indirectly.

It has been shown that AD converters in MCI group had a lower fluorodeoxyglucose uptake in the precuneus [20]. The distribution of the atrophy region in correlation with the BF-227 uptake value of the precuneus differed greatly from that in the inferior temporal gyrus since there was no correlation between the atrophy of precuneus and the lateral temporal lobe. The occipitotemporal area shows atrophy in AD. However, why correlation was detected within a relatively confined area in the occipitotemporal region is unclear, and further studies are needed to clarify this issue.

The results of the BF-227 uptake analyses suggest that areas such as the inferior temporal gyrus show a different pathological mechanism than the precuneus. According to Braak and Braak [21], BF-227 retention pattern of amyloid deposits was different between the precuneus and the inferior temporal gyrus. Because the inferior temporal gyrus showed faster amyloid deposition than the precuneus in AD subjects, the consequent atrophy of the inferior temporal gyrus may have occurred faster.

By contrast, in the symptomatic theory, as for cognitive function, the inferior temporal gyrus, in complex form, such as a face, has been reported to be associated with the recall of long-term and short-term memory that is semantic memory to the stimulation of visual information based on experience [22]. The precuneus is associated with the recall of episodic memory, and has a particularly important role in remembering a visual image associated with the memory [23]. Although the semantic memory and the episodic memory are both classified as long-term memory, it is known that episodic memory changes are observed at an early stage of dementia. On the contrary, changes in semantic memory are observed in severe cases of dementia. In the future, one should consider employing tasks such as the delayed recall task (logical memory) of Wechsler memory scale-revised test (WMS-R), which evaluates episodic memory to associate the degree of amyloid deposition and atrophy in the precuneus. Besides, as an indication of semantic memory, educational history should be considered. To elucidate pathological changes involved in semantic and episodic memory impairments, it may be necessary to consider educational history and inferior temporal gyrus. Dore *et al*. [24] reported that a significant reduction in cortical thickness in the precuneus and the hippocampus was associated with episodic memory impairment in the NC PiB-positive (NC+) group when compared with the NC− group. Even in our study, the atrophy of the right precuneus in NC group correlated to the BF-227 uptake in inferior temporal gyrus. BF-227 uptake in the inferior temporal gyrus in healthy elderly subjects is a result that suggests the possibility of episodic memory impairment. Further studies including the examination of cognitive function on our samples will be necessary.

Whether amyloid deposition evidenced by BF-227 uptake causes neuropathy is imperfectly understood. The amyloid does not necessarily become the effective biomarker of AD development. Petersen *et al*. [25] reported that a considerable number of subjects had biomarkers of Aβ deposition and neurodegeneration inconsistent with the proposed AD model. For example, 29% of Mayo Clinic Study of Aging (MCSA) subjects and 17% of Alzheimer’s disease neuroimaging initiative 1 (ADNI-1) subjects showed evidence of neurodegeneration without amyloid deposition. However, the results of this study suggest that BF-227 uptake in the inferior temporal gyrus may be used as a biomarker of AD development. In the area where BF-227 uptake was shown, we found that there was the area that may have become the biomarker of the development of AD and it may not become so.

In this study, the changes observed apply only to AD subjects that fulfill the diagnostic criteria of AD and not to MCI. Recently, the possibility that the cytotoxic load of the tau protein (a tau hypothesis) affects the onset of the dementia the most was suggested [26]. In NC, BF-227 uptake of the inferior temporal gyrus may be related to the atrophy of the precuneus at a preclinical state. Alternatively, these results may have shown a false correlation that actually reflected the cytotoxicity through the tau. Performing tau imaging would be imperative to understand this mechanism in detail.

Group comparisons of gray matter atrophy using MR imaging demonstrated the most advanced atrophy in AD, followed by MCI, and then by NC. Group comparisons of Aβ deposition using PET imaging showed no significant differences in BF-227 uptake between MCI and AD. The reason could be that Aβ deposition already reached a plateau at a stage of MCI. Significant differences were observed between AD and NC, as well as between MCI and NC. Correlation analyses between local Aβ deposition and gray matter atrophy showed that in AD, the Aβ deposition in the inferior temporal gyrus was strongly related to the gray matter atrophy in the inferior temporal gyrus itself. In addition, the atrophy of the inferior temporal gyrus was also caused by Aβ deposition in various areas (middle temporal gyrus, middle occipital gyrus, supramarginal gyrus and angular gyrus). On the contrary, Aβ deposition in the precuneus was related to the gray matter atrophy of right occipitotemporal areas. Therefore, in AD, pathologic influences of Aβ deposition on local atrophy may be region-dependent. In addition, in NC, Aβ deposition in the inferior temporal gyrus correlated to precuneus atrophy.

This report represents the first study using amyloid PET with BF-227 and MRI, in addition to evaluating the correlation between local Aβ deposition and local gray matter atrophy. A pilot study was used with small size, and replication of this study using additional samples will be required to confirm our findings.

This study was supported in part by research grants from Grants-in-Aid for Scientific Research (C), Ministry of Education, Culture, Sports, Science and Technology, Japan. The research theme was ‘Study on diagnosis of early Alzheimer’s disease by β-amyloid imaging (BF-227)’ (20591470).

## Acknowledgements

**Conflicts of interest**

There are no conflicts of interest.
